# Construction and validation of a personalized risk prediction model for in-hospital mortality in patients with acute myocardial infarction undergoing percutaneous coronary intervention

**DOI:** 10.1016/j.clinsp.2025.100580

**Published:** 2025-02-01

**Authors:** Bing-Zheng Xu, Bin Wang, Jian-Ping Chen, Jin-Gang Xu, Xiao-Ya Wu

**Affiliations:** The Wenzhou Medical College Dongyang Hospital Emergency Department, Zhejiang, China

**Keywords:** Myocardial infarction, Risk factors, Prognosis, Nomogram model

## Abstract

**Background:**

Although emergency Percutaneous Coronary Intervention (PCI) has been shown to reduce mortality in patients with Acute Myocardial Infarction (AMI), the risk of in-hospital death remains high. In this study, the authors aimed to identify risk factors associated with in-hospital mortality in AMI patients who underwent PCI, develop a nomogram prediction model, and evaluate its effectiveness.

**Methods:**

The authors retrospectively analyzed data from 1260 patients who underwent emergency PCI at Dongyang People's Hospital between June 1, 2013, and December 31, 2021. Patients were divided into two groups based on in-hospital mortality: the death group (*n* = 61) and the survival group (*n* = 1199). Clinical data between the two groups were compared. The Least Absolute Shrinkage and Selection Operator (LASSO) regression was used to select non-zero coefficients of predictive factors. Multivariable logistic regression analysis was then performed to identify independent risk factors for in-hospital mortality in AMI patients after emergency PCI. A nomogram model for predicting the risk of in-hospital mortality in AMI patients after PCI was constructed, and its predictive performance was evaluated using the c-index. Internal validation was performed using the bootstrap method with 1000 resamples. The Hosmer-Lemeshow test was used to assess the goodness of fit, and a calibration curve was plotted to evaluate the model's calibration.

**Results:**

LASSO regression identified d-dimer, B-type natriuretic peptide, white blood cell count, heart rate, aspartate aminotransferase, systolic blood pressure, and the presence of postoperative respiratory failure as important predictive factors for in-hospital mortality in AMI patients after PCI. Multivariable logistic regression analysis showed that d-dimer, B-type natriuretic peptide, white blood cell count, systolic blood pressure, and the presence of postoperative respiratory failure were independent factors for in-hospital mortality. A nomogram model for predicting the risk of in-hospital mortality in AMI patients after PCI was constructed using these independent predictive factors. The Hosmer-Lemeshow test yielded a Chi-Square value of 9.43 (*p* = 0.331), indicating a good fit for the model, and the calibration curve closely approximated the ideal model. The c-index for internal validation was 0.700 (0.560‒0.834), further confirming the predictive performance of the model. Clinical decision analysis demonstrated that the nomogram model had good clinical utility, with an area under the ROC curve of 0.944 (95 % CI 0.903‒0.963), indicating excellent discriminative ability.

**Conclusion:**

This study identified B-type natriuretic peptide, white blood cell count, systolic blood pressure, d-dimer, and the presence of respiratory failure as independent factors for in-hospital mortality in AMI patients undergoing emergency PCI. The nomogram model based on these factors showed high predictive accuracy and feasibility.

## Highlights

• Construction of a personalized risk prediction model for in-hospital mortality in patients with acute myocardial infarction undergoing Percutaneous Coronary Intervention (PCI).
• Identification of risk factors associated with in-hospital mortality in AMI patients who underwent PCI.
• Development of a nomogram prediction model for in-hospital mortality in AMI patients after emergency PCI.
• Evaluation of the nomogram model's effectiveness through c-index, internal validation, Hosmer-Lemeshow test, and calibration curve analysis.

## Introduction

Acute Myocardial Infarction (AMI), encompassing ST-segment Elevation Myocardial Infarction (STEMI) and Non-ST-segment Elevation Myocardial Infarction (NSTEMI), is characterized by myocardial ischemic necrosis resulting from coronary artery occlusion. Plaque rupture in the coronary arteries is the main cause of AMI, with risk factors including smoking, hypertension, diabetes, and hyperlipidemia.^1^^,^^2^ Despite advancements in treatment, AMI remains a leading cause of global mortality.^3^ Percutaneous Coronary Intervention (PCI) is the primary treatment for AMI, particularly for STEMI and AMI with cardiogenic shock, effectively reducing mortality rates.^4^, ^5^, ^6^ However, the mortality rate of AMI remains high.^7^^,^^8^ To improve patient outcomes and enhance physician-patient communication, clinical guidelines recommend assessing the prognostic risk of patients with myocardial infarction.^9^^,^^10^

Currently, commonly used methods for prognostic assessment during hospitalization after emergency PCI include the Global Registry of Acute Coronary Events (GRACE) and Thrombolysis in Myocardial Infarction (TIMI) scoring systems.^11^, ^12^, ^13^ However, these scoring systems are not specifically designed for patients undergoing emergency PCI, and traditional methods only differentiate the risk level without providing a specific assessment of mortality risk. Additionally, novel indicators associated with patient prognosis, such as pro-brain natriuretic peptide^14^ and d-dimer,^15^ have emerged but are underutilized in traditional scoring systems.

With advancements in computational capabilities, researchers have proposed new risk assessment models to aid clinical risk evaluation. Nevertheless, these models have limitations. In 2019, US researchers developed a model to predict in-hospital mortality risk in post-PCI patients based on the New York PCI Reporting System and machine learning capabilities. However, this model encompassed all PCI patients, including elective surgery patients, and lacked comprehensive variables, thereby affecting its performance.^16^ In 2020 and 2022, prediction models for in-hospital mortality risk in STEMI patients after PCI were published. However, these models included a large number of variables, rendering them cumbersome and limiting their clinical application.^17^^,^^18^ Furthermore, subjective judgments and the exclusion of recently used clinical blood markers compromised the predictive accuracy of these models. Lastly, these studies solely focused on STEMI patients and neglected other patients requiring emergency PCI.

In summary, there are several deficiencies in the current prognosis assessment of patients after emergency PCI, including scoring systems and models. An effective approach to evaluate the prognosis of these patients during hospitalization remains unclear.^19^^,^^20^ In this study, the authors retrospectively analyzed the clinical characteristics of 1260 patients undergoing emergency PCI and constructed a nomogram model to predict in-hospital mortality risk in patients with acute myocardial infarction after emergency PCI. This model provides valuable insights for assessing patient conditions and guiding treatment decisions in clinical settings.

## Methods and materials

### Study population

The authors retrospectively analyzed the clinical data of patients who underwent emergency Percutaneous Coronary Intervention (PCI) at Dongyang People's Hospital between June 1, 2013, and December 31, 2021. The inclusion criteria were as follows: 1) Patients diagnosed with acute myocardial infarction, confirmed by meeting at least two of the following criteria: (a) Presence of symptoms such as chest tightness, chest pain, or atypical symptoms like syncope, nausea, vomiting, or upper abdominal pain; (b) Electrocardiogram findings consistent with acute myocardial infarction, such as ST-segment elevation or depression, or new-onset complete left bundle branch block; (c) Elevated cardiac enzymes, with troponin or creatine kinase-MB levels greater than or equal to 2 times the normal value. 2) Patients who underwent emergency PCI, defined as immediate surgical intervention following the diagnosis of myocardial infarction. Exclusion criteria were as follows: 1) Patients under 18-years of age; 2) Patients with aortic dissection; 3) Patients with acute pericarditis; 4) Patients with acute myocarditis; 5) Patients who died within 24 h of hospitalization; 6) Patients confirmed to have coronary myocardial bridging after PCI.

### Data collection

Demographic and clinical data of patients with acute myocardial infarction upon admission were collected using the hospital's electronic medical record system. This included information on gender, age, smoking and drinking history, as well as initial measurements of respiratory rate, heart rate, creatine kinase-MB, creatine kinase, high-sensitivity C-reactive protein, creatinine, pro-brain natriuretic peptide, platelet count, troponin-T, total bilirubin, aspartate aminotransferase, white blood cell count, d-dimer, triglycerides, high-density lipoprotein, low-density lipoprotein, total cholesterol, albumin, prealbumin, apolipoprotein-A, apolipoprotein-B, magnesium, red blood cell hematocrit, left ventricular ejection fraction, systolic blood pressure, diastolic blood pressure, as well as the occurrence of respiratory failure (defined as an oxygenation index less than 300 mmHg) and the type of culprit vessel (non-ST-segment elevation myocardial infarction).

### Establishment and validation of the nomogram model

After collecting patient data, a thorough data cleaning and analysis process was conducted. Missing data were imputed using the mice package in R software. Univariate analysis was performed to identify significant variables for lasso regression analysis. For continuous variables identified through lasso regression analysis, the linearity assumption was assessed using the box Tidwell function. Variables with a p-value less than 0.05 were considered to have no linear relationship with the logit. Multicollinearity among significant variables was assessed using Variance Inflation Factors (VIFs), with VIFs less than 10 indicating no significant multicollinearity. After excluding variables with linear relationships and significant multicollinearity, logistic regression and stepwise regression analysis were conducted to identify independent risk factors for model construction and nomogram development. The discrimination, calibration, and clinical utility of the model were evaluated, and internal validation was performed using the bootstrap method. Discrimination of the model was assessed by the Area Under the Curve (AUC), with an AUC greater than 0.75 indicating good discriminative ability. Calibration of the model was evaluated by constructing a calibration plot, and a high degree of overlap between the fitted curve and the standard curve indicated good predictive accuracy, with a p-value greater than 0.05 indicating good goodness-of-fit. The clinical utility of the model was evaluated using Decision Curve Analysis (DCA), with the net benefit represented on the vertical axis and the predicted risk of disease on the horizontal axis. Comparison of the model curve (mod) with the all curve and none curve (representing two extreme curves) revealed greater clinical significance and net benefit when there was a significant difference between the model curve and the two extreme curves. Model validation was conducted using the bootstrap method for internal validation, with *B* = 1000 repetitions. The predictive ability of the model was evaluated by comparing the calibration curve with the ideal curve.

### Statistical analysis

Skewed measurement units were represented using the median and interquartile range [M (P25, P75)]. Categorical variables were analyzed using the Chi-Square (χ^2^) test and presented as percentages. All statistical analyses were performed using R software to ensure accurate and reliable results.

## Results

### Distribution of missing data

**Fig. 1:**
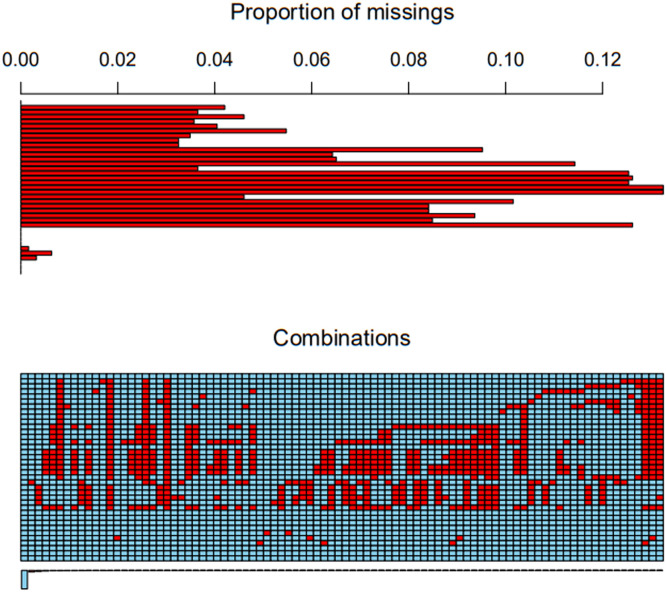
Distribution of missing data.

The distribution plot reveals that the missing values for individual variables are all below 15 %, indicating the feasibility of performing multiple imputations. Further details can be found in Fig. 1.

### Basic characteristics and univariate analysis of the study population

A total of 1260 patients, comprising 301 females and 959 males, were enrolled in this study. Among them, 61 deaths occurred, resulting in a mortality rate of 4.8 %. Univariate analysis demonstrated significant differences (*p* < 0.05) between the survival and death groups in various parameters, including troponin-T, creatine kinase isoenzyme, d-dimer, B-type natriuretic peptide, aspartate aminotransferase, C-reactive protein, creatinine, white blood cell count, total bilirubin, albumin, prealbumin, hematocrit, creatine kinase, left ventricular ejection fraction, systolic blood pressure, diastolic blood pressure, heart rate, respiratory rate, age, smoking history, gender, and respiratory failure. However, no statistically significant differences (*p* > 0.05) were observed between the two groups in terms of platelet count, magnesium, high-density lipoprotein, low-density lipoprotein, total cholesterol, apolipoprotein-A, apolipoprotein-B, triglycerides, left anterior descending artery, right coronary artery, other blood vessels, alcohol consumption history, and non-ST segment elevation. Further details can be found in Table 1.

**Table 1:** Comparison of general clinical data between the survival and death groups.

Variable	T	Survival group (*n* = 1199)	Death group (*n* = 61)	*p*
Troponin T	2.82 (0.75, 7.16)	2.63 (0.68, 6.64)	10 (3.09, 10)	<0.001
Creatine kinase isozyme	119 (45, 276.25)	114 (44, 258)	279 (78, 637)	<0.001
D-dimer	0.75 (0.38, 1.19)	0.71 (0.37, 1.13)	3.62 (1.66, 7.81)	<0.001
Type B forebrain diuretic opeptide	1280 (526.9, 3388.75)	1182 (497, 2945.5)	15,165 (7125, 33,181)	<0.001
Glutamic-oxalacetic transaminase	453 (269, 801.25)	437 (266.5, 768.5)	1118 (525, 1684)	<0.001
C-reactive protein	4.8 (1.64, 15.2)	4.52 (1.6, 14.24)	25.11 (7.1, 90.48)	<0.001
Creatinine	78 (66, 96)	77 (65, 94)	128 (92, 247)	<0.001
Leucocyte count	10.82 (8.47, 13.83)	10.65 (8.31,13.44)	17.2 (13.41, 21.38)	<0.001
Platelet count	220 (183, 261)	219 (184, 260)	231 (175, 295)	0.294
Magnesium	0.86 (0.81, 0.91)	0.86 (0.81, 0.91)	0.86 (0.81, 0.93)	0.295
Total bilirubin	14.4 (10.7, 18.82)	14.5 (10.8, 18.9)	13.3 (8.3, 17.3)	0.046
Albumin	36.8 (34.4, 39.1)	36.9 (34.7, 39.3)	33.9 (30.7, 36)	<0.001
Prealbumin	208 (173.95, 245.08)	213 (177.6, 247)	163 (135, 195.2)	<0.001
Hematocrit	0.4 (0.36, 0.43)	0.4 (0.37, 0.44)	0.37 (0.32, 0.4)	<0.001
HDL	0.97 (0.82, 1.15)	0.97 (0.83, 1.15)	1 (0.77, 1.25)	0.505
LDL	2.66 (2.12, 3.32)	2.66 (2.13, 3.32)	2.71 (1.94, 3.51)	0.92
Cholesterol total	4.37 (3.72, 5.14)	4.36 (3.72, 5.13)	4.55 (3.56, 5.21)	0.814
Apolipoprotein A	0.95 (0.83, 1.11)	0.95 (0.83, 1.11)	0.95 (0.8, 1.14)	0.614
Apolipoprotein B	0.88 (0.7, 1.06)	0.88 (0.7, 1.06)	0.89 (0.66, 1.09)	0.744
Creatine kinase	1101.5 (348.75, 2619.5)	1067 (341, 2513.5)	2557 (560, 5356)	<0.001
Left heart ejection fraction	55 (46, 60)	55 (47, 60)	45 (40, 54)	<0.001
Systolic pressure	97.5 (88, 109)	98 (89, 110)	82 (66, 92)	<0.001
Diastolic pressure	57 (50, 66)	57 (50, 66)	49 (40, 55)	<0.001
Heart rate	95 (84, 106)	94 (84, 105)	120 (104, 132)	<0.001
Breathing rate	20 (20, 24)	20 (20, 23)	30 (22, 34)	<0.001
Glycerin trilaurate	1.38 (1.04, 1.96)	1.38 (1.03, 1.97)	1.46 (1.19, 1.81)	0.372
Age	65 (53, 76)	65 (53, 75)	78 (72, 86)	<0.001
Left anterior descending branch				0.108
No	694 (55 %)	667 (56 %)	27 (44 %)	
Yes	566 (45 %)	532 (44 %)	34 (56 %)	
Right coronary artery				0.141
No	897 (71 %)	848 (71 %)	49 (80 %)	
Yes	363 (29 %)	351 (29 %)	12 (20 %)	
Other blood vessels				0.132
No	883 (70 %)	846 (71 %)	37 (61 %)	
Yes	377 (30 %)	353 (29 %)	24 (39 %)	
History of smoking				<0.001
No	522 (41 %)	480 (40 %)	42 (69 %)	
Yes	674 (53 %)	657 (55 %)	17 (28 %)	
Once upon a time, now quit	64 (5 %)	62 (5 %)	2 (3 %)	
History of drinking				0.051
No	662 (53 %)	623 (52 %)	39 (64 %)	
Yes	564 (45 %)	545 (45 %)	19 (31 %)	
Once upon a time, now quit	34 (3 %)	31 (3 %)	3 (5 %)	
Sex				<0.001
Man	959 (76 %)	930 (78 %)	29 (48 %)	
Woman	301 (24 %)	269 (22 %)	32 (52 %)	
Non-ST elevation for MI				0.46
No	1018 (81 %)	966 (81 %)	52 (85 %)	
Yes	242 (19 %)	233 (19 %)	9 (15 %)	
Failure of respiration				<0.001
No	1211 (96 %)	1167 (97 %)	44 (72 %)	
Yes	49 (4 %)	32 (3 %)	17 (28 %)	

### Lasso regression for variable selection

**Fig. 2:**
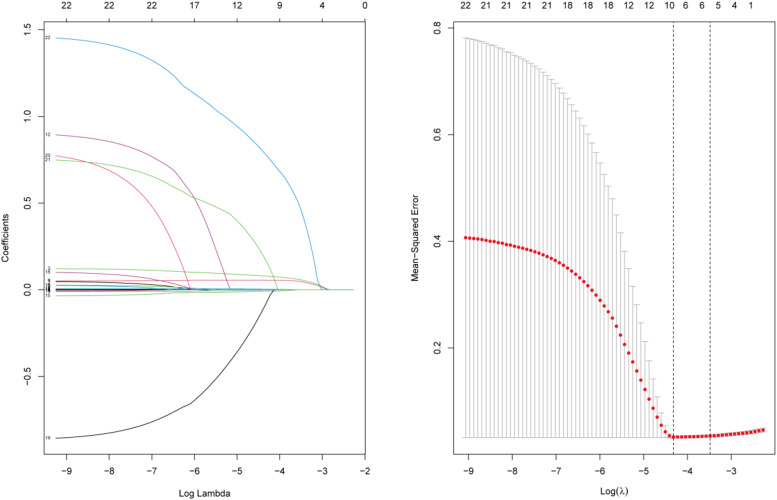
Predictor plots identified through Least Absolute Shrinkage and Selection Operator (LASSO) regression analysis.

To identify the key clinical and laboratory parameters associated with in-hospital mortality following Percutaneous Coronary Intervention (PCI) in patients with Acute Myocardial Infarction (AMI), the authors employed Lasso regression analysis. In this analysis, the dependent variable was whether death occurred during hospitalization, categorized as NO (0) or YES (1). Through Lasso regression dimensionality reduction, the authors selected 35 variables as potential independent variables. To determine the optimal value of the regularization parameter (λ), the authors employed 10-fold cross-validation and calculated the λ-values. Ultimately, the authors selected the λ-value within one standard deviation range of the minimum mean square prediction error as the optimal value. After performing Lasso regression analysis, the authors identified 7 predictor factors with non-zero coefficients from the initial set of 35 variables. These factors included d-dimer, B-type natriuretic peptide, white blood cell count, heart rate, aspartate aminotransferase, systolic blood pressure, and the presence of postoperative respiratory failure (Fig. 2).

### Multivariable logistic regression analysis

To investigate the potential predictors of in-hospital mortality in patients after emergency Percutaneous Coronary Intervention (PCI), a multivariable logistic regression model was constructed using Lasso regression. The independent variables included d-dimer (measured value), systolic blood pressure (measured value), the presence of postoperative respiratory failure (NO = 0, YES = 1), B-type natriuretic peptide (measured value), white blood cell count (measured value), heart rate (measured value), and aspartate aminotransferase (measured value). The results of the analysis revealed that d-dimer, B-type natriuretic peptide, white blood cell count, and the presence of postoperative respiratory failure were identified as independent risk factors, systolic blood pressure was identified as an independent protective factor for in-hospital mortality in patients after emergency PCI (*p* < 0.05) (Table 2).

**Table 2:** Multivariable logistic regression and stepwise regression analysis.

Variable	Logistic Regression analysis		Stepwise regression analysis	
	OR (95 % CI)	*p*	OR (95 % CI)	*p*
D-dimer	1.108 (1.032‒1.185)	0.003	1.104 (1.028‒1.180)	0.005
Type B forebrain diuretic opeptide	1.214 (1.127‒1.335)	<0.001	1.331 (1.211‒1.421)	<0.001
Glutamic-oxalacetic transaminase	1.000 (0.999‒1.000)	0.153	1.000 (0.999‒1.001)	0.144
Leucocyte count	1.068 (1.001‒1.139)	0.046	1.078 (1.012‒1.148)	0.020
Systolic pressure	0.974 (0.956‒0.993)	0.007	0.970 (0.953‒0.987)	<0.001
Heart rate	1.009 (0.996‒1.023)	0.176	NA	NA
Whether there is respiratory failure	2.863 (1.133‒6.954)	0.022	2.744 (1.085‒6.677)	0.029

### Construction of nomogram

**Fig. 3:**
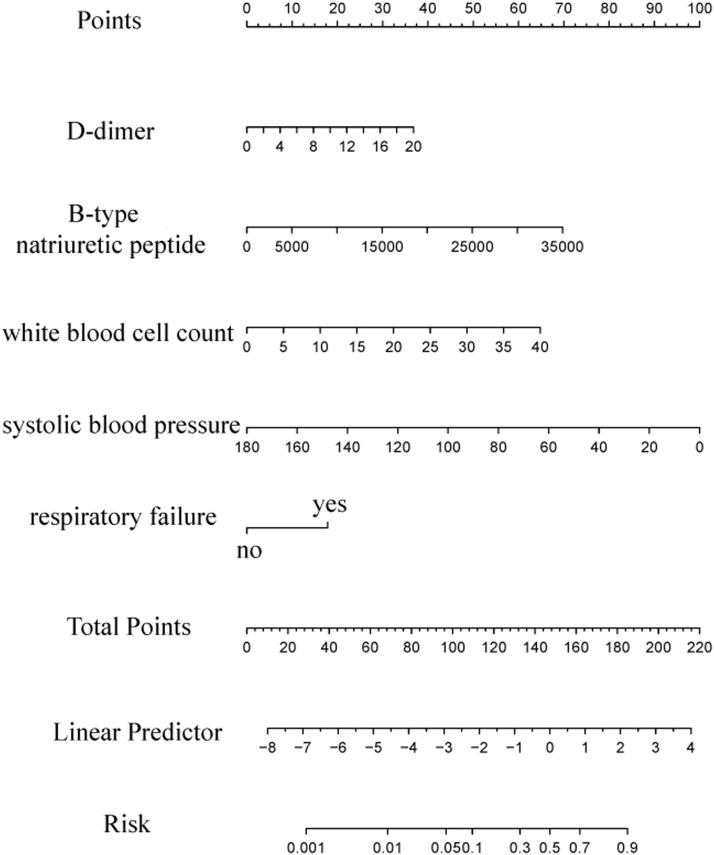
Nomogram predicting in-hospital mortality in patients after emergency PCI for acute myocardial infarction.

To predict in-hospital mortality in patients after emergency Percutaneous Coronary Intervention (PCI) for acute myocardial infarction, the authors constructed a nomogram based on the predictor variables. The detailed construction is illustrated in Fig. 3. The nomogram incorporates specific values of each indicator, which correspond to corresponding scores positioned above the graph. By summing up these scores, the total score is obtained. The total score, in turn, corresponds to the specific risk of death indicated below the graph. Notably, the line graph visually demonstrates that patients with higher total scores exhibit a greater likelihood of experiencing in-hospital mortality.

### Validation of the nomogram model

**Fig. 4:**
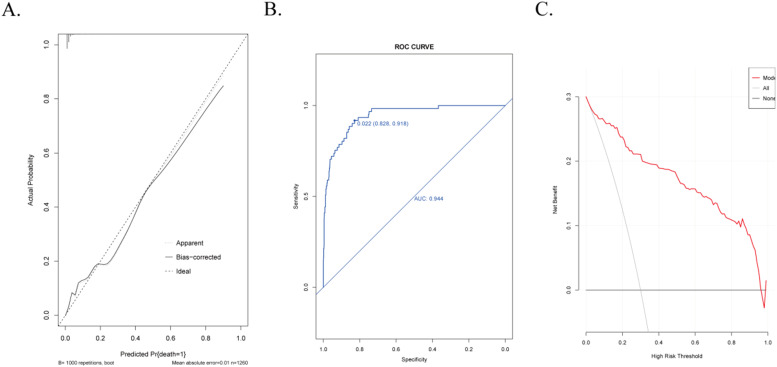
Validation of the nomogram model. (A) Calibration plot, (B) ROC curve, (C) DCA curve.

Internal validation was conducted using bootstrapping with 1000 samples to assess the robustness of the prediction model. The results demonstrated a Harrell C-Index of 0.700 (95 % Confidence Interval [95 % CI 0.560‒0.834]), indicating excellent calibration of the derived model. Furthermore, the Hosmer-Lemeshow test chi-square value was 9.43, with a corresponding p-value of 0.331, suggesting high predictive accuracy. The area under the Receiver Operating Characteristic (ROC) curve was 0.944 (95 % CI 0.903‒0.963), reflecting the outstanding discriminatory power of the model. Notably, the Decision Curve Analysis (DCA) revealed that the model surpassed the two extreme curves, underscoring its remarkable clinical predictive performance (Fig. 4).

## Discussion

Acute Myocardial Infarction (AMI) is a pervasive cause of mortality among individuals with cardiovascular diseases.^21^^,^^22^ Emergency Percutaneous Coronary Intervention (PCI) has emerged as the preferred treatment strategy for patients experiencing Acute ST-segment elevation Myocardial Infarction (AMI), with mounting evidence supporting its ability to enhance the prognosis of AMI patients.^4^^,^^23^ However, certain patients, such as those afflicted with AMI complicated by cardiogenic shock or malignant arrhythmias, continue to face a substantial risk of in-hospital mortality, despite the utilization of mechanical support devices like the Intra-Aortic Balloon Pump (IABP) and percutaneous cardiopulmonary support.^24^^,^^25^ Numerous national and international registries have consistently reported a persistently high mortality risk for AMI patients.^26^^,^^27^ Consequently, the objective of this investigation was to explore the pertinent risk factors associated with in-hospital mortality in AMI patients following PCI, and to develop a nomogram model utilizing selected predictive factors, thereby furnishing clinicians with a valuable tool for assessing patient condition and guiding treatment decisions.

This study employed multiple logistic regression analysis to identify independent risk factors associated with in-hospital mortality in patients undergoing emergency Percutaneous Coronary Intervention (PCI). The variables examined included d-dimer, B-type natriuretic peptide, white blood cell count, and postoperative respiratory failure. Furthermore, systolic blood pressure was identified as an independent protective factor. These findings have significant clinical implications. d-dimer is a widely utilized clinical marker that exhibits elevated levels in individuals at a higher risk of thromboembolic events, such as recurrent myocardial infarction and pulmonary embolism.^15^^,^^28^ As a degradation product of fibrin, increased d-dimer levels reflect disruptions in blood coagulation and fibrinolysis, serving as an indicator of thrombus formation and hemorheological abnormalities in patients with acute myocardial infarction. The results of this study demonstrate that elevated d-dimer is an independent risk factor for in-hospital mortality in patients undergoing emergency PCI, emphasizing the critical role of thrombus formation and hemorheological abnormalities in patient mortality. Elevated d-dimer levels may signify the presence of thrombus formation and hemorheological abnormalities, which can lead to complications such as coronary restenosis and thromboembolism, thereby increasing the risk of patient mortality.

B-type Natriuretic Peptide (BNP) is a cardiac peptide hormone secreted by the heart, serving as a reliable biomarker for increased cardiac load and myocardial injury. Its levels have been found to accurately reflect the severity of heart failure and are closely associated with the NYHA functional class.^29^ During episodes of acute heart failure, there is a sharp surge in plasma BNP levels. BNP levels are strongly linked to left ventricular systolic dysfunction,^30^^,^^31^ making them sensitive indicators for assessing ventricular function, heart failure severity, treatment efficacy, and prognosis.^32^^,^^33^ In patients with heart failure, BNP levels significantly rise in proportion to ventricular function impairment and cardiac injury severity. A recent meta-analysis has demonstrated a significantly heightened risk of adverse prognosis in patients with elevated BNP levels following acute myocardial infarction, underscoring the value of BNP measurement in risk stratification for these patients.^14^ The findings of this study suggest that elevated BNP levels independently contribute to in-hospital mortality risk in patients after emergency Percutaneous Coronary Intervention (PCI), highlighting the substantial impact of increased cardiac load and myocardial injury on patient survival. The elevation of BNP levels can reflect augmented cardiac load and myocardial injury, potentially leading to complications such as heart failure and arrhythmias, thereby escalating the risk of patient mortality.

Elevated white blood cell count is a commonly observed marker of inflammation, which can arise from various factors including stress, reperfusion injury, or infection. Prior investigations have demonstrated a correlation between increased white blood cell count and diminished salvage of myocardial cells in individuals undergoing Percutaneous Coronary Intervention (PCI), suggesting a potential link to unfavorable prognosis.^34^ The current study further substantiates the independent role of elevated white blood cell count in the risk of in-hospital mortality following emergency PCI, underscoring the criticality of the inflammatory response in patient survival. The elevation of white blood cell count serves as a reflection of the systemic inflammatory response magnitude, which may contribute to the development of complications such as systemic inflammatory response syndrome and infection, thereby augmenting the risk of patient mortality. Consequently, vigilant monitoring and effective management of white blood cell count assume paramount clinical significance in the post-emergency PCI care of patients.

The occurrence of postoperative respiratory failure serves as a clinical manifestation that effectively reflects the pulmonary function and overall health status of patients. The findings of this study demonstrate that the presence of postoperative respiratory failure independently contributes to the risk of in-hospital mortality among patients undergoing emergency Percutaneous Coronary Intervention (PCI). This highlights the crucial role of pulmonary function and overall health status in patient mortality. The emergence of postoperative respiratory failure often signifies severe cardiac injury, which subsequently leads to pulmonary edema and impaired gas exchange, ultimately resulting in systemic hypoxia and an elevated risk of mortality.^35^ Consequently, it becomes imperative to enhance respiratory support therapy and systemic support therapy for patients experiencing postoperative respiratory failure, with the aim of improving oxygenation levels and averting potential complications.

Previous studies have demonstrated that the blood pressure upon admission serves as a crucial determinant of the in-hospital prognosis for patients diagnosed with Acute Myocardial Infarction (AMI). Specifically, patients with concomitant hypertension and AMI exhibit a notably poorer prognosis,^36^ whereas other investigations have established a significant association between lower levels of systolic and diastolic blood pressure upon admission and an increased risk of in-hospital mortality.^37^^,^^38^ In the present study, the authors have observed a statistically significant disparity in systolic blood pressure upon admission between the deceased and survival groups, with the former exhibiting lower levels. The authors postulate that this decrease in systolic blood pressure may impede myocardial perfusion, consequently exerting an adverse impact on prognosis. The subsequent decline in systolic blood pressure postoperatively among patients may be attributed to severe cardiac injury and diminished ejection capacity. Hypotension signifies compromised myocardial perfusion, which can potentially lead to multi-organ dysfunction or even failure, thereby amplifying the mortality risk.

A predictive model utilizing Lasso regression and multiple logistic regression analysis was developed to assess the risk of in-hospital mortality following emergency Percutaneous Coronary Intervention (PCI). The model incorporated variables including d-dimer, B-type natriuretic peptide, white blood cell count, systolic blood pressure, and occurrence of postoperative respiratory failure. Remarkably, the model exhibited excellent predictive accuracy and discrimination. Internal validation and Decision Curve Analysis (DCA) further validated the model's performance. Internal validation yielded a Harrell C-Index of 0.700, indicating satisfactory calibration. Moreover, DCA demonstrated that the model surpassed the two extreme curves, underscoring its clinical predictive ability. Consequently, this model offers precise prognostication of in-hospital mortality risk in patients undergoing emergency PCI. Discrimination of the model was verified by an area under the Receiver Operating Characteristic (ROC) curve of 0.944, signifying its ability to effectively differentiate between survivors and non-survivors. Notably, the discrimination achieved by this model outperformed previous studies.^39^ For instance, the AUC values for TIMI and GRACE scores in short-term prognosis evaluation of NSTEMI patients were merely 0.54 and 0.77 for STEMI patients, respectively. Similarly, the AUC of the GRACE score in short-term prognosis evaluation for NSTEMI patients was 0.80 and 0.82 for STEMI patients. Additionally, other novel models achieved AUC values ranging from 0.78 to 0.85. Importantly, these aforementioned scoring systems and models failed to consider the clinical net benefit and lacked comprehensive evaluation. In contrast, the present model excelled in discrimination and overall evaluation compared to these existing scoring systems and models. The improved performance of this model can be attributed to the incorporation of novel indicators such as B-type natriuretic peptide precursor and d-dimer. These indicators, not routinely employed in clinical practice, hold significant implications for the evaluation of patients following emergency PCI.

This study possesses several limitations. Firstly, it is a single-center study with a relatively limited sample size, which may introduce selection bias and inadequate statistical power. Therefore, it is imperative to conduct further multicenter studies with larger sample sizes to validate the findings of this study. Secondly, the predictive variables considered in this study were solely clinical data and laboratory parameters, potentially excluding other significant risk factors from the model. Hence, additional research is warranted to explore and incorporate other potential risk factors. Lastly, this study solely focuses on the risk of in-hospital mortality in patients undergoing emergency Percutaneous Coronary Intervention (PCI), necessitating further investigations for long-term follow-up and prognosis assessment.

## Conclusion

In summary, the present study reveals that several independent factors, including d-dimer, B-type Natriuretic Peptide (BNP), Systolic Blood Pressure (SBP), White Blood Cell Count (WBC), and postoperative respiratory failure, significantly impact in-hospital mortality in patients undergoing emergency Percutaneous Coronary Intervention (PCI) for Acute Myocardial Infarction (AMI). To provide clinicians with an effective tool for early risk assessment, the authors have developed a nomogram model incorporating these factors. The nomogram exhibits excellent discriminative ability, calibration, and clinical utility, making it a valuable resource for predicting in-hospital mortality risk in AMI patients after PCI.

## Availability of data and materials

The datasets used and/or analyzed during the current study are available from the corresponding author upon reasonable request.

## Ethical statement

This study was conducted in accordance with the ethical principles outlined in the Helsinki Declaration and its amendments. Approval was obtained from the Ethics Committee of Dongyang People's Hospital (approval number Dongren Medical 2022-YX-037), and written consent was obtained from the patients or their family members. All data were analyzed anonymously, and personal information was completely removed to ensure confidentiality.

## CRediT authorship contribution statement

**Bing-Zheng Xu:** Conceptualization, Methodology, Project administration, Resources, Writing – original draft, Writing – review & editing, Investigation, Validation. **Bin Wang:** Investigation, Validation. **Jian-Ping Chen:** Investigation, Validation. **Jin-Gang Xu:** Investigation, Validation. **Xiao-Ya Wu:** Conceptualization, Methodology, Project administration, Resources, Writing – original draft, Writing – review & editing.

## Declaration of competing interest

The authors declare no conflicts of interest.
